# Occupational exposures and cancer risk in commercial laundry and dry cleaning industries: a scoping review

**DOI:** 10.1186/s12889-023-17306-y

**Published:** 2023-12-21

**Authors:** Emma Ann Landskroner, Candace Su-Jung Tsai

**Affiliations:** Department of Environmental Health Sciences, Fielding School of Public Health, University of California, 650 Charles E. Young Drive S., MC 177220, 90095-1735, 90095-1735 Los Angeles, California United States

**Keywords:** Cancer, Dry cleaning industry, Hazardous chemical exposure, Indoor solvent exposure, Occupational exposures

## Abstract

**Background:**

The laundry and dry cleaning industries are critical for maintaining cleanliness and hygiene in our daily lives. However, they have also been identified as sources of hazardous chemical exposure for workers, leading to potentially severe health implications. Despite mounting evidence that solvents like perchloroethylene and trichloroethylene are carcinogenic, they remain commonly used in the industry. Additionally, while alternative solvents are increasingly being utilized in response to indications of adverse health and environmental effects, there remains a significant gap in our understanding of the potential risks associated with exposure to these new agents.

**Methods:**

This study aims to identify gaps in the literature concerning worker exposure to contemporary toxic chemicals in the laundry and dry cleaning industry and their associated carcinogenic risks. A scoping review of peer-reviewed publications from 2012 to 2022 was conducted to achieve this objective, focusing on studies that detailed chemical exposures, sampling methods, and workers within the laundry and dry cleaning sector.

**Results:**

In this scoping review, 12 relevant papers were assessed. A majority (66%) examined perchloroethylene exposure, with one notable finding revealing that biomarkers from dry cleaners had significant micronuclei frequency and DNA damage, even when exposed to PCE at levels below occupational exposure limits. Similarly, another study supported these results, finding an increase in early DNA damage among exposed workers. Separate studies on TCE and benzene presented varied exposure levels and health risks, raising concern due to their IARC Group 1 carcinogen classification. Information on alternative solvents was limited, highlighting gaps in health outcome data, exposure guidelines, and carcinogenic classifications.

**Conclusion:**

Research on health outcomes, specifically carcinogenicity from solvent exposure in dry cleaning, is limited, with 66% of studies not monitoring health implications, particularly for emerging solvents. Further, findings indicated potential DNA damage from perchloroethylene, even below set occupational limits, emphasizing the need to reevaluate safety limits. As alternative solvents like butylal and high-flashpoint hydrocarbons become more prevalent, investigations into the effects of their exposure are necessary to safeguard workers’ health. This scoping review is registered with the Open Science Framework, registration DOI: 10.17605/OSF.IO/Q8FR3.

**Supplementary Information:**

The online version contains supplementary material available at 10.1186/s12889-023-17306-y.

## Background

The laundry and dry cleaning industry in the United States comprises approximately 36,000 facilities employing 157,400 workers regularly using solvents and hazardous chemicals for deep cleaning and stain removal [1–4]. The International Labour Organization (ILO) indicates that workers predominantly encounter solvents through inhalation, with acute, high levels leading to delirium, respiratory depression, and death, and chronic low levels being associated with cancer, reproductive issues, and neurotoxicity [3, 5, 6]. In addition, pollutants generated from these operations have been linked to harmful environmental impacts, including air pollution and groundwater contamination, resulting in secondary prospective routes of exposure [3, 5, 6].

Current evidence suggests that dry cleaning and laundry workers are at an increased risk of cancer mortality [7, 8]. A National Institute for Occupational Safety and Health (NIOSH) study involving 1,708 dry-cleaners exposed to solvents like perchloroethylene (PCE) had a significant excess of total cancer deaths (271 deaths, Standardized Mortality Ratio (SMR) of 1.25, 95% Confidence Interval (CI) (1.11–1.41)) [7]. Further analysis revealed statistically significant SMR results for tongue, bladder, esophagus, intestine, lung, and cervix cancer, with tongue cancer and ischemic heart disease elevated among individuals solely exposed to PCE [7]. Similarly, another study found an exposure-response relationship between solvent and bladder cancer, kidney cancer, heart disease, and, separately, non-Hodgkin’s lymphoma [9]. Other research has found that dry cleaners are at increased risk for head and neck squamous cell carcinoma, especially with PCE and trichloroethylene (TCE) exposure [10, 11].

Despite compelling evidence of its adverse health outcomes and efforts to regulate and reduce its prevalence, PCE remains the industry standard [12, 13]. In response to growing concern over the use of harmful solvents, the industry has begun to explore new and more ecologically sound options with unknown human health implications. As a result, individuals in the dry cleaning industry remain at risk of exposure to already established carcinogenic substances, like PCE and TCE, and emerging solvents with unknown, carcinogenic health effects.

### Known exposures to traditionally toxic agents

TCE and PCE, common solvents classified as volatile organic compounds (VOCs) (defined in Supporting Information (SI) VOC Section), are widely used chemicals in the dry cleaning industry [5]. These non-flammable and colorless solvents are effective stain removers commonly used in the US and Europe [11, 12]. Based on extensive epidemiological evidence, the International Agency for Research on Cancer (IARC) has classified PCE as probably carcinogenic to humans (Group 2A) and TCE as definitively carcinogenic to humans (Group 1), linking exposure to increased cancer risks [5, 12]. PCE exposure is associated with liver, kidney, and central nervous system damage, as well as an elevated risk of bladder cancer and non-Hodgkin lymphoma [5, 14–17]. Comparatively, there’s substantial human evidence demonstrating a positive association between TCE exposure and kidney cancer, as well as an increased risk of non-Hodgkin’s lymphoma, cervical cancer, and liver cancer, while in vivo data indicates TCE induces tumor development in the liver, lungs, testes, and hematopoietic tissue [5, 18–21].

## Substitution with “safer” alternatives

As increasing evidence is published substantiating PCE and TCE’s adverse health and environmental impacts, regulatory agencies worldwide, such as the United States Environmental Protection Agency (EPA) and the European Union, through Registration, Evaluation, Authorization, and Restriction of Chemicals (REACH) regulations, have taken steps to phase out or restrict their use [22–28]. Their rules aim to promote the adoption of safer solvents and alternative cleaning methods.

The Toxic Use Reduction Institute (TURI) assessed alternative options to PCE in dry cleaning, ranking substances based on technical, economic, environmental, regulatory, and health factors [29]. The alternatives were graded from one to five, with one being the most desirable: (1) Wet cleaning (water and detergent without solvents); (2) Liquid carbon dioxide (used with specialized detergents under the pressure of 700 PSI); (3) High flashpoint hydrocarbons, propylene glycol ethers, and butylal; (4) Siloxane; (5) N-propyl bromide [12, 29].

The safest alternatives, wet cleaning and liquid carbon dioxide, among the five substitution options, are non-solvent based. However, adopting these methods has been slow due to multiple drawbacks (i.e., fabric deterioration, expensive machinery, and labor-intensive) [12, 30]. Consequently, solvent-based methods remain the most prevalent approach for cleaning [12].

### High-flashpoint hydrocarbons

High-flashpoint hydrocarbons, Group 3 by TURI, are aliphatic hydrocarbons and are volatile petroleum-based solvents with a flashpoint at or above 140 °F (60 °C) [29, 31, 32]. Manufactured under several trade names, including DF-2000™ (ExxonMobil Corporation) and EcoSolv® (Cheveron Phillips Chemical Company, LLC), high-flashpoint hydrocarbons are generally less volatile and are not designated hazardous air pollutants (HAPs) or ozone-depleting substances (ODS) [31, 33, 34]. While these solvents have gained popularity in the US, being touted as a greener alternative to PCE, there is still limited evidence on the potential carcinogenicity of exposure and no occupational exposure limit (OEL) or biological exposure indices (BEI) to reference [14, 32].

### Propylene glycol ethers (PGE)

Propylene Glycol Ether (PGE) solvents, Group 3 by TURI, are organic, volatile, water-soluble compounds with a flash point ranging from 160 to 212 °F (71–100 °C ) [29, 35]. There are numerous types of PGE solvents available for dry cleaning; some of the most well-known formulations are glycol n-butyl (DPnB) and dipropylene glycol tert-butyl (DPtB), which have emerged as promising substitutes for PCE and TCE [36, 37]. While certain PGEs are carcinogenic, DPnB and DPtB have not been linked to any adverse environmental or health impacts [36, 37]. These biodegradable solvents are characterized by their low volatility and efficient cleaning properties [12, 36, 37]. Specific PGE solvents have established OELs, but the IARC has not classified PGEs for carcinogenicity [29].

### Butylal

Butylal, known as dibutoxymethane, Group 3 by TURI, is a combustible liquid with a flash point of 144 °F (62 °C), and is commonly used in the dry cleaning industry, primarily as SolvonK4 ^TM^ (Kreussler Inc.). It contains butylal (> 99% purity) and small amounts of n-butanol and formaldehyde [29, 38, 39]. Limited data is available on the health effects of butylal, with most studies focusing on dermal and oral exposures. No OELs or BEIs have been established, and the IARC has not reviewed its carcinogenicity [29, 39].

### Siloxane

Decamethylcyclopentasiloxane, or D5, Group 4 by TURI, is colorless and odorless volatile methyl siloxane used as a solvent in the GreenEarth® dry cleaning system [12, 29]. Made of a combustible modified liquid silicone with a flashpoint of 170 °F (76.6 °C), D5 is a less aggressive cleaner than PCE. It has been identified as a more environmentally friendly alternative by the California Office of Environmental Health Hazard Assessment [29, 40]. The IARC has not classified D5, as there is insufficient data examining its toxicity and there are currently no established OELs or BEIs for D5 [29, 40].

### n-Propyl bromide

N-Propyl Bromide (n-PB), or 1-bromopropane, Group 5 by TURI, is a volatile chemical similar to PCE and other halogenated hydrocarbon solvents, with differing flash points depending on the testing method [29]. It was promoted as an alternative to PCE in the EU via REACH and later determined to be a “regrettable substitution.” The United States EPA has since added n-PB to the Clean Air Act list of hazardous pollutants, as exposure causes irritation, neurologic effects, and possible damage to the nervous system [29, 41, 42]. There are currently OELs but no BEIs; the IARC has classified n-PB as possibly carcinogenic to humans (Group 2B) [41, 43, 44].

## Rationale

Exposure to hazardous chemicals, such as volatile organic compounds, solvents, and other agents in the laundry and dry cleaning industry, threatens human health. Despite their widespread use and implementation of new evolving chemical compositions, there is still a limited understanding of the specific cancers and chronic diseases that may be associated with these substances. Given the industry’s historical association with carcinogenic compounds, existing evidence of higher rates of cancer among dry cleaners, and the potential carcinogenic effects of newer alternatives, carcinogenicity was deemed to be the primary health effect of concern. A scoping review was determined to be the most appropriate method to evaluate worker exposure in the laundry and dry-cleaning industry and identify gaps related to both traditional and emerging solvents, their potential toxicity, cancer risks, and other health implications.

## Main text

### Methods

The scoping review was conducted in accordance with the Joanna Briggs Institute methodology for scoping reviews [45].The primary and secondary research questions guiding this review aimed to identify toxic substances in the laundry and dry cleaning industry, their potential links to cancer, gaps pertaining to emerging solvents, their potential toxicity, carcinogenic risks, and any other health implications. Studies of various designs were included and charted in tables to ensure a thorough analysis. A Reporting Items for Systematic Reviews checklist - extension for Scoping Reviews (PRISMA-ScR) was used to guide the steps followed in this scoping review [46].

### Search strategy

The search strategy was designed to identify relevant peer-reviewed articles related to occupational exposures in the laundry and dry cleaning industry and any associated cancers. To achieve this objective, an initial limited search was performed on several reputable databases. The search terms used were based on the words and phrases found in the titles and abstracts of relevant articles, which were then used to formulate a comprehensive search strategy. This process ensured that all relevant studies were identified and included in the review.

Search terms were developed based on three primary categories: occupational-related terms, exposures of interest-related terms, and one outcome of interest term (SI Table S2). A general search algorithm was developed and adapted for each included database. A systematic search of peer-reviewed literature published in English between January 1, 2012, and December 1, 2022, was performed via PubMed, Science Direct, NIH Library, Embase, EBSCOhost, and Google Scholar. The focus on literature between 2012 and 2022 was intended to capture the most recent advancements and exposure profiles.

The population of interest comprised laundry and dry cleaning workers. The exposure scope was limited to relevant and commonly used chemicals throughout the dry cleaning process. The outcome of interest was exposure concentration of airborne chemicals and any information available regarding the potential adverse human health outcomes, specifically cancer development due to exposure. Relevant studies of various designs, such as risk assessments, cohort studies, case studies, and biomonitoring studies, were included in the search strategy. Overview articles, commentary, editorial, or opinion articles were excluded to ensure the reliability and quality of the review’s findings.

### Screening of articles

**Fig. 1:**
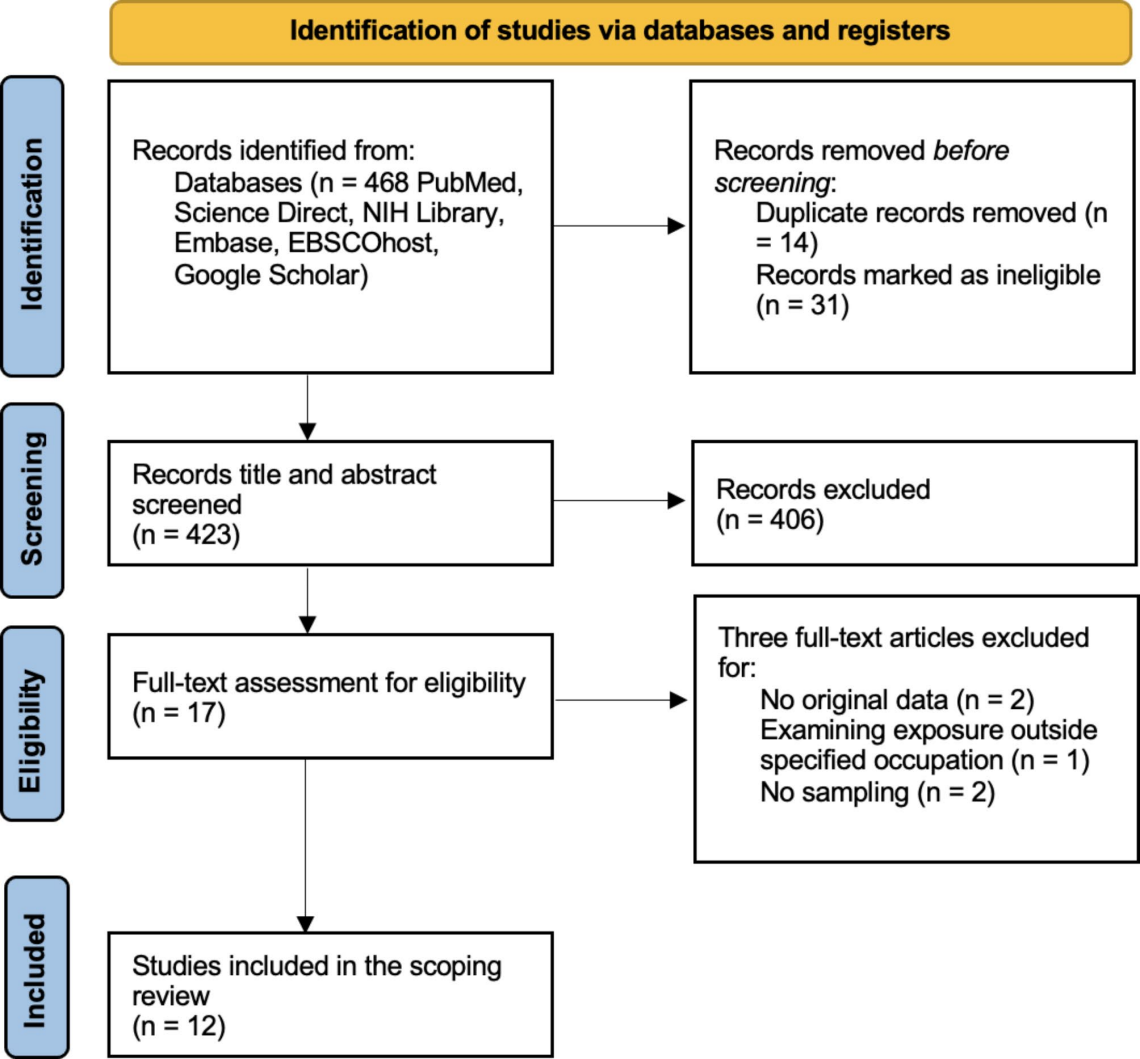
PRISMA Flow Diagram for the screening and selection of articles

Articles were retrieved from the first systematic search and uploaded into EndNote 20.5 (SI Table S3). Duplicate articles and ineligible articles were identified and removed. The remaining articles’ titles and abstracts were independently screened against the specified inclusion and exclusion criteria (SI Table S1). Articles that did not meet the criteria were excluded. All articles that did meet the requirements were pulled for a full-text review. The remaining articles were approved and included in the scoping review (SI Table S4). The discussed screening and selection process is presented via Version 1 PRISMAs Flow Diagram (Fig. 1).

### Data extraction

The primary author (EL) extracted data from the selected articles. The data extracted included the first authors’ names, country of origin, the number of workplaces, the total number of workers, exposure measurement conditions, exposure agents of interest, measured outcomes, and reported human health impacts if specified (i.e., cancer). The indication “NA” was marked if data was unavailable. In studies where sampling was conducted, outcomes were grouped by sampling type, i.e., Area Air Sampling, Personal Air Sampling, and Biological Sampling. When appropriate, subcategories were used to indicate when multiple measurements and sampling methods were used within a single study.

Data on area air or personal air concentrations in ppm used in some studies were converted to mg/m³ as follows [47]:

$$Concentration\,mg/m\, = \,\left( {ppm} \right) \times \left( {molecular\,weight} \right) \times \left( {1/{{24.45}^ * }} \right)$$

*Molar volume of gas at 1 atmosphere and 25 °C (unless another temperature was specified in the study, noted in charting).

Data on airborne concentration found in the included studies were compared with current OELs from OSHA, ACGIH, and NIOSH. Data on biological concentration found in the included studies were compared with current BEIs set by the ACGIH. “NA” was used to designate when no OEL or BEI was available for an examined substance.

## Results

A PRISMA flow chart (Fig. 1) summarizes the screening and selection process. From an initial pool of 468 articles, 12 were identified for the scoping review after eliminating duplicates (n = 14), ineligible articles (n = 31), and articles that did not fit the specified criteria (n = 411). The 12 included studies, presented in Table 1, were conducted in ten countries across the globe, four reporting area sampling only (n = 4: 1 Various VOCs (Nonane, Decane, Undecane, Nonanal, Decanal, O-xylene, and Toluene); 1 TCE; 1 PCE; 1 TCE and PCE), one reporting personal and area sampling (n = 1: 1 butylal and high-flashpoint hydrocarbons (DF-2000)), four reporting personal and biological sampling (n = 4: 3 PCE; 1 PCE and trichloroacetic acid (TCA)), and three reporting biological sampling only (n = 3: 2 PCE; 1 benzene). Details summarizing the study location, design, number of workplaces and workers involved, and exposure measurement conditions are provided in Table 1. For OEL and BEI details of each examined substance, refer to Table S4 in the SI.

**Table 1:** Overview of study characteristics: location, design, sample size, and exposure measurement conditions

Author	Country/ Year Pub.	Study Type	Number of workplace and workers	Exposure Measurement Conditions
Ceballos et al. [39]	USA/ 2016	Cross-Sectional	4 dry cleaning shops/not reported	Personal and Area Sampling: At least two days evaluating each shop. Full-shift and short-term area air sampling. Duration of machine dry cleaning 70–80 min. No temp. reported.
Eun et al. [59]	South Korea/ 2022	Cross-Sectional	1 laundry facility/not reported	Area Sampling: 1 kg of cotton fiber washed with petroleum solvent. Experiment performed 3x. Duration of machine dry cleaning 23 min. Avg. temp 20 °C at 1 atm.
Friesen et al. [82]	China/ 2015	Retrospective Survey	Not reported/not reported	Area Sampling: Database measurments recorded 932 short-term (≤ 20 min.) measurments collected across different occupations. 23 samples collected in the laundry industry. Limited data on sample and analytic method. Temp. at 25 °C at 1 atm.
Habib et al. [50]	UAE/ 2018	Cross-Sectional	4 dry cleaning shops/not reported	Area Sampling: Air sampler set up at three positions in each facility. Samples were collected for three 8 h. workdays. No temp. reported.
Sadeghi et al. [55]	Iran/ 2013	Cross-Sectional	10 dry cleaning shops/not reported	Area Sampling: Four samples taken at each dry cleaning shop - one sample every 15 days. No temp. reported.
Dias et al. [62]	Brazil/ 2017	Cross-Sectional and Biomonitoring	24 dry cleaning shops, one electroplating industry, one research laboratory, one automotive paint shop/25 workers	Personal and Biological Sampling: Samples collected at the end of 8 h. work shift in breathing zone of workers for 15 min. post collection of exhaled air samples. Mean indoor temp. 24 ± 3 °C.
Everatt et al. [66]	Lithuania/ 2013	Case-Control	Six dry cleaning shops/59 volunteers (30 exposed and 29 non-exposed)	Personal and Biological Sampling: Breathing zone samples collected on two consecutive workdays in 150-min. intervals post 8 h. shifts. Avg. temp. 23 °C.
Lucas et al. [53]	France/ 2015	Cross-Sectional	22 dry cleaning shops/145 volunteers (50 exposed and 95 non-exposed)	Personal and Biological Sampling: Personal passive samplers placed for a full work-shift on a single day. No temp. reported.
Modenese et al. [61]	Italy/ 2019	Cross-Sectional and Biomonitoring	21 dry cleaning shops/60 workers	Personal and Biological Sampling: Personal passive samplers worn for a full 8 h. Work-shift on a single day. No temp. reported.
Azimi et al. [65]	Iran/ 2017	Case-Control	Not reported/59 volunteers (33 exposed and 26 non-exposed)	Biological Sampling (Peripheral Blood and Comet Assay): Peripheral blood samples collected in the morning from each participant. Cells counted in each comet slide and analyzed.
Shim et al. [64]	South Korea/ 2013	Case-Report	One dry cleaning shop/2 workers	Biological Sampling (Urine and Blood): After showing symptoms of jaundice, subjects were admitted to the hospital, where blood and urine samples were taken for laboratory work.
Ziener & Braunsdorf [63]	Germany/ 2014	Case-Control and Biomonitoring	One dry cleaning shop/14 volunteers (4 exposed and 10 non-exposed)	Biological Sampling (Exhaled Air): End-exhaled air sampling collected a week prior to last shift of working week. Two samples were collected consecutively. Subjects filled sampling tubes for approx. 4 min. Avg. temp. 20 °C.

Table 2 summarizes each study organized by the substance analyzed, sampling method, measured quantitative outcomes and reported biological health effects discussed in the paper. The IARC classification for each chemical has been included in Table 2 to serve as a direct tool to gauge the current understanding of carcinogenic potential and identify substances that warrant further investigation.

**Table 2:** Summary of laundry and dry cleaning exposures: outcomes, reported biological impacts, and IARC classifications

Exposure Agent of Interest	Measured Outcomes #	Reported Biological Health Effects and IARC Classification
PCE [50] Area Air Sampling	Facility A: 52.28-1,356* ► Facility B: 47.47–508.7* ► Facility C: 20.34-3,458* ► Facility D: 7.798-1,627* ►	NHEM IARC 2 A
PCE [62] Personal Air Sampling	0.014–3.205* Median: 0.599*	NHEM IARC 2 A
Biological Sampling [62]	Exhaled Air: 0.006–2.635* Median: 0.325*	
PCE [66] Personal Air Sampling	0.0-77.46 Mean (SD): 31.40 (23.51)	Significant increase in micronuclei and DNA damage compared to controls. No significant difference in CA. The frequency of exposure and employment duration was associated with CA frequency. IARC 2 A
Biological Sampling [66] Comet Assay	CA/100 cells: Chromosome-type aberrations (SD): 1.04 (0.66) Chromatid breaks (SD): 2.03 (1.68)	
Comet Assay [66]	MN/1000 cells: Total MN mean (SD): 11.36 (6.90)	
Comet Assay [66]	Comet tail length in peripheral blood lymphocytes: Mean (SD): 10.45 (6.52) µm	
PCE [53] Personal Air Sampling	1.5–221 ► Mean: 47.41 Median: 25.5	No increased rate of clinical symptoms or acute exposure symptoms related to PCE. IARC 2 A
Biological Sampling [53]	Blood: 11.8–544 µg/l Mean125.9 µg/l	
PCE [61] Personal Air Sampling	0.1–86.0 Mean (SD): 17.0 (18.5)	NHEM IARC 2 A
Biological Sampling [61]	Exhaled Air: 0.1–37.4 ►Mean (SD): 10.4 (10.3)	
Biological Sampling [61]	Urine: 0.1–40.0 µg/l Mean (SD): 8.4 µg/l (11.7)	
PCE [65] Biological Sampling Comet Assay	Tail length: 6.63–67.2 μm Median: 25.85 μm DNA% in tail: 5.73–48.85 Median: 23.03 Tail moment: 0.42–44.29 Median: 7.07	Greater DNA damage in exposed dry cleaners compared to non-exposed. Evidence of genotoxic and carcinogenic effects. IARC 2 A
PCE [63] Biological Sampling	Exhaled Air: 3.4–16.7* Mean: 9.35*	NHEM IARC 2 A
PCE [55] Area Air Sampling	42.70–516.0* ^ ►Mean: 110.9*∩ Max: 960.0* ★	NHEM IARC 2 A
TCA [61] Biological Sampling	Urine: 0.02–3.2 µg/l Mean (SD): 0.7 µg/l (0.9)	NHEM IARC 2B
TCE [55] Area Air Sampling	29.50-543.7* ^ ► Mean: 95.69*★∩ Max: 964.0* ★	NHEM IARC 1
TCE [82] Area Air Sampling	AM: 710.0 ★ GM: 570.0 ★ GSD: 2.000 Max: 2,200 ★	NHEM IARC 1
Butylal [39] Area Air Sampling	Full shift: 0.02557-2.032* Short-term: 1.115–12.46*	NHEM IARC N/A
Personal Air Sampling [39]	Full shift: 0.1114-5.440* Task-based: 2.753–12.45*	
High-flashpoint hydrocarbons (Df-2000) [39] Area Air Sampling	Full shift: 0.1600–5.600 Short-term: 5.300–37.00	NHEM IARC N/A
Personal Air Sampling [39]	Full shift: 0.990–5.400 Task-based: <3.800–7.900	
VOCs [59] Area Air Sampling	Total POCP: 33.7ppm Nonane: 74.28* Decane: 68.33* Undecane: 29.80* O-xylene: 8.185*	NHEM IARC 3 for xylene and toluene
	Total SOAF: 8.3ppm Xylene: 10.08* Decane: 13.36* Undecane: 13.49* Nonane: 4.163* Toluene: 1.018*	
Benzene [64] Biological Sampling	Subject A: Urine phenol-benzene: 2.489 mg/g creatinine t.t-munoic acid-benzene: 0.058 mg/g creatinine	Jaundice and subsequent diagnosis of late-stage gallbladder cancer. IARC 1
	Subject B: Urine phenol-benzene: 12.895 mg/g creatinine t.t-munoic acid-benzene: 0.057 mg/g creatinine	

SD = Standard deviation; AM = Arithmetic mean; GM = Geometric mean; GSD = Geometric standard deviation; # = All concentrations reported in mg/m³ unless otherwise specified; * = Concentrations were reported in a different unit and calculated in mg/m³ as given in the methods section; ★ = Concentration above at least one OEL value; ► = Maximum concentration range value outside at least one of the presented OEL values; ^ = Mean range; ∩ = Grand mean; POCP: Photochemical Ozone Creation Potential; SOAF: Secondary Organic Aerosol Formation; NHEM = No health effects monitored in the study, NA = Not Available

IARC 2 A is Group 2 A, Probably carcinogenic to humans [5]

IARC 2B is Group 2B, Possibly carcinogenic to humans [79]

IARC 1 is Group 1, Carcinogenic to humans [5]

IARC 3 is Group 3, Unclassifiable as to carcinogenicity in humans [80]

The sampling methods of the included studies encompass area air sampling, measurements of indoor static air pollution at a fixed location indicative of potential work area exposure; personal air sampling, conducted within the worker’s breathing zone [48]; and biomonitoring, employed to assess exposure to chemicals via internal dose [49]. These measurements can then be compared with relevant OELs and BEIs to evaluate exposure risk (SI Table S4). In Table 2, a star symbol designates measured values that exceed at least one established OEL (OSHA, ACGIH, or NIOSH) or BEI (ACGIH) for the analyzed substance. Measurements with a maximum range value exceeding any OEL or BEI are marked with a right-pointed triangle.

### Exposure studies

For studies measuring air concentrations, the duration of measurements ranged from 15 min. to 8 h, with one study (Sadeghi et al. 2013) only specifying that samples were taken every 15 days without indication of the sampling time and another study not including the sampling methodology (Friesen et al. 2015).

Of the nine studies reporting some form of air sampling, four reported at least one measurement exceeding OEL standards (Habib et al. 2018; Lucas et al. 2015; Sadeghi et al. 2013; Friesen et al. 2015). Habib et al. 2018 measured area air sampling at four different facilities in three working positions where PCE concentration ranged from 1.15-510 ppm (7.798–3,458 mg/m³) [50]. Positions involving unloading clothing demonstrated the highest maximum concentration of PCE exposure across three of the four shops, recording above OSHA’s permissible exposure limit-time weighted average (PEL-TWA) at 100 ppm (300 mg/m³) [51]. All four shops presented values exceeding ACGIH threshold limit value-time weighted average (TLV-TWA) of 25 ppm (169.5 mg/m³), indicating high levels of exposure; however, no biological health effects were measured [50, 52].

Lucas et al. 2015 also sampled for PCE exposure, collecting personal air and peripheral blood samples from 50 exposed employees at 22 shops and comparing measurements with non-exposed individuals. Air sampling showed a mean concentration of 47.41 mg/m³, ranging between 1.5 and 221 mg/m³, with the maximum value exceeding ACGIHs TLV-TWA [53]. Blood analysis revealed an average PCE concentration of 125.9 µg/L (0.1259 mg/L) with a range of 11.8–544 µg/L (0.0118–0.544 mg/L). Eight percent of workers had PCE levels exceeding 400 µg/L (0.400 mg/L), falling either close to or outside ACGIH BEI of 0.5 mg/L [52–54]. While there was no direct link between work hours and symptoms, 78% of exposed workers reported potential PCE-related symptoms, predominantly neurological effects (87%) [53].

Sadeghi et al. 2013 conducted area air sampling for PCE and TCE at ten dry cleaning shops, with supplemental sampling collected from a gas station, underground soil, and effluent. The grand mean for the dry cleaning shops PCE was 110.9 µg/L (110.9 mg/m³), with the maximum value of 960 µg/L (960 mg/m³) [55]. One facility exceeded OSHA’s PEL-TWA and ACGIH TLV-TWA, reporting 320–960 µg/L (320–960 mg/m³). The grand mean for TCE was 95.69 µg/L (95.69 mg/m³), and a maximum measurement of 964 µg/L (964 mg/m³), higher than ACGIHs TLV-TWA of 10 ppm (54 mg/m³) [55]. Although there were samples that exceeded exposure values, human health effects were not measured amongst workers in this study [55, 56].

Friesen et al. 2015. conducted a retrospective survey study examining TCE exposure via short-term air samples from the laundry and dry cleaning industry between 1976 and 1977. From 23 samples, the arithmetic mean was 710 mg/m³, with a maximum of 2,000 mg/m³. All measurements exceeded ACGIHs TLV-TWA as well as OSHAs at 100 ppm (535 mg/m³), and NIOSH recommended exposure limit-time weighted average (REL-TWA) at 25 ppm (134.3 mg/m³), and no health outcomes were examined [56–58]. It is essential to consider that these measurements were taken in the late 1970s, likely before implemented regulations, and thus may not be representative of current exposure levels.

Of the other air sampling studies, Ceballos et al. 2016 conducted personal and area sampling analyzing butylal and the high-flashpoint hydrocarbon DF-2000 in four dry cleaning shops. Full-shift personal exposure levels for DF-2000 varied from 0.99 to 5.4 mg/m³, while full-shift personal exposure to butylal ranged from 0.017 to 0.83 ppm (0.1114-5.440 mg/m³) [39]. Task-based exposure, especially near dry cleaning machines or during fabric pressing, was higher, aligning with what was seen in Habib et al. 2018’s study, that workspace location influences exposure risk—currently, both butylal and high-flashpoint hydrocarbon lack OELs, BEIs, and IARC classifications to compare to. Further, no health effects were monitored in this study.

Eun et al. 2022 conducted area sampling and examined various VOCs in one laundry facility, focusing on 77 analytes. Photochemical ozone creation penitential (POCP) was estimated via a method proposed by Derwent et al. [59, 60]. The secondary organic aerosol formation potential (SOAP) was calculated by multiplying the emissions by the degree to which the compound produces SOA in the presence of additional mass concentration relative to the SOA formed when the same amount is present [59]. Results showed that 61% of the substances were detected with nonane, decane, undecane, and o-xylene, contributing to 95% of the potential. Of all the chemicals detected, three (nonane, o-xylene, and toluene) have OELs, with all reported measurements under OEL values. Eun et al. 2022, also conducted a risk assessment considering hazard identification and dose-response assessments for carcinogenic and non-carcinogenic compounds. The observed carcinogens had a mean total estimated cancer risk of 2.36 × 10^− 5^, nitrobenzene having the highest cancer risk (1.26 × 10^− 4^), and acrylonitrile, carbon tetrachloride, nitrobenzene, bromodichloromethane, and chloromethane exceeding standards [59]. Of the non-carcinogenic substances, the mean total hazard quotient was 1.19, with bromomethane having the highest risk index at 5.95, and bromomethane, chlorobenzene, o-xylene, and heptachlor-1,3-butadiene exceeding standards, indicating that even those substances that are not identified as carcinogenic, may still pose health risks [59].

Modenese et al. 2019 conducted personal air sampling and biological sampling via exhaled air and urine concentration measurements for PCE and TCA in 21 dry cleaning shops and 60 workers. For PCE, the mean personal passive sample concentration was 17.0 mg/m³ (SD: 18.5 mg/m³), under OEL values, but had a maximum PCE range value of 37.4 mg/m^3^, outside of the ACGIH BEI of 3ppm (20.34 mg/m^3^) [54]. The mean exhaled alveolar air was 10.4 mg/m³ (SD: 10.3 mg/m³), with a range of 0.1–32.4 mg/m³ at the end of shift, and urine concentration was 8.4 µg/L (0.0084 mg/L) [61]. ACGIH BEI is available for exhaled air collected before the start of the shift at 3 ppm (20.34 mg/m³) [54, 61]. Most exhaled air samples were within the range of established BEI; however, a few exceeded the set value [61]. TCA concentrations were only measured via urine sampling, with the mean concentration at 0.7 mg/L (SD: 0.9 mg/L), below ACGIH BEI for TCA at 15 mg/L [54].

Dias et al. 2017 conducted personal and biomonitoring exhaled air sampling in 24 dry cleaning facilities measuring PCE exposure. The study references that all personal air samples collected from the shops, with one exception, had PCE concentrations exceeding the inhalation reference concentration (IRC) recommended by the EPA of 0.016 mg/m³; however, the concentrations did not exceed the previously referenced OEL standards [62]. Personal sampling results ranged from 14.0 to 3,205 µg/m³ (0.014–3.205 mg/m³) [62]. Exhaled air of exposed individuals had concentrations ranging from 6.0 to 2,635 µg/m³ (0.006–2.635 mg/m³) with a median concentration of 325 µg/m³ (0.325 mg/m³) within ACGIH BEI for exhaled air [62] Associated health impacts were not monitored throughout this study. Additional sampling was taken in an electroplating facility, research laboratory, and automotive paint preparation shop, with the highest reported concentrations from the dry cleaning facilities and their workers [62].

Ziener & Braunsdorf, 2014, collected biological sampling via end-exhaled breath in one dry cleaning shop with four workers and ten controls [63]. Samples were collected one day before the working shift, twice consecutively [63]. PCE concentrations in the exposed group ranged from 3.4 to 16.7 µg/L (3.4–16.7 mg/m³), with a mean of 9.35 µg/L (9.35 mg/m³) within the established ACGIH BEI for in end exhaled air (20.34 mg/m³) [54, 63]. No health outcome assessment information was collected during this study.

Shim et al. 2013 studied two elderly individuals who worked in a small dry cleaning shop for 40 years, exposed to dry cleaning solvents, specifically benzene [64]. Both were admitted to the hospital for jaundice. Benzene metabolite tests revealed the male’s levels were 12.895 mg/g creatine for phenol and 0.057 mg/g creatine for t,t-muconic acid; the females were 2.489 mg/g and 0.058 mg/g, respectively. Both patients’ benzene levels remained within the standard range for urine phenol-benzene (< 50 mg/g creatine, 10ppm standard) and t,t-muconic acid-benzene (< 1 mg/g creatine, 10ppm standard) [64]. Neither patient exceeded the ACGIH BEI value for t,t-muconic acid in urine of 500 µg/g creatinine [54]. A likely explanation could be that benzene is broken down by the body over time, with a biological half-life of approximately 24 h [64].

### Health effects studies

Three studies (Azimi et al. 2017, Everatt et al. 2013, Shim et al. 2013) explored biomarkers of health effects, identifying discernible and measurable biological alterations due to chemical exposure. Azimi et al. 2017  examined 33 dry cleaners and 26 controls, conducting comet assays on peripheral blood lymphocyte samples to assess the potential genotoxic and carcinogenic effects of PCE exposure. Fifty cells were counted on each comet slide and evaluated by comet assay parameters (TL, %DNA in tail, TM, and olive TM). Results found a significant increase in early DNA damage among the exposed individuals vs. the non-exposed, as primary DNA damage to leukocytes in dry cleaners was high (exposed median tail length: 25.85 vs. non-exposed: 5.61; exposed median %DNA in the tail: 23.03 vs. non-exposed: 8.77; exposed median tail moment: 7.07 vs. non-exposed: 1.03) [65]. However, no correlation was determined between the duration of employment and DNA damage [65].

Everatt et al. 2013 measured for PCE, collecting personal air samples and peripheral blood, sampling from 59 volunteers (30 exposed dry cleaning workers and 29 controls) [66]. The mean concentrations in personal air samples were 31.40 mg/m³ and ranged from 0 to 77 mg/m³, within the established OSHA and ACGIH OELs [66]. Similar to Azimi et al. 2017 findings, dry cleaners had higher MN frequency (MN/1000 binucleated cells) and DNA damage, measured by comet tail length compared to the control group (10.45 vs. 5.77, P < 0.05) [66]. Additionally, there was a significant association between chromosome aberration (CA) frequency, employment duration, and frequency of exposure, as well as increased micronuclei (MN) damage in workers compared to controls (CA:1.04 vs. 0.59, P = 0.005; MN: 11.36 vs. 6.96, P < 0.05; DNA: 10.45 vs. 5.77, P = 0.05). No significant relationship was observed between these effects and the level of PCE exposure sampled. However, the differences between these groups were significant, indicating that levels below the established OELs could still cause genotoxic damage to the body [66].

Shim et al. 2013, as also discussed in Sect. 5.1. reported laboratory findings from two patients exposed to benzene, with the first patient, a 60-year-old man, having abnormal laboratory findings (total/direct bilirubin: 18.4/9.9 mg/dL; AST/ALT 183/331 IU/L; ALP 700 IU/L; GGT 537 IU/L; CA19-9 of 4,980 U/mL) [64]. The patient was diagnosed with stage IV gallbladder cancer. The second patient, a 60-year-old female, also had abnormal laboratory results (total/direct bilirubin: 9.8/6.4 mg/dL; AST/ALT 172/497 IU/L; ALP 411 IU/L; GGT 1,304 IU/L; CA19-9 of 613 U/mL) [64]. Like the male, the female patient was diagnosed with metastasized gallbladder cancer [64]. Both patients presented evidence of liver damage and toxicity, indicative of long-term benzene exposure. Neither patient had elevated risk factors for gallbladder cancer compared to the general population [64, 67].

## Discussion

The data presented in Table 2 reveals a concerning trend; of the 12 papers examined, only four (33%) were assessed for health effects, either through clinical symptoms or biomarkers of exposure. Three of these four (75%) focused on PCE, an already classified Group 2A carcinogen. The remaining one (25%) examined benzene, a Group 1 carcinogen. This indicates two major gaps in the literature: (1) The bulk of the studies investigating exposure within the dry cleaning and laundry industry seem to sidestep crucial study design components that, if incorporated, would allow for a more holistic picture of the health risks associated with exposure to these substances. This omission includes the measurement of exposure biomarkers to ascertain the internal dose of the investigated solvent and the examination of biomarkers related to biological effects, fundamental to determining human response to these compounds, including their carcinogenic effects. (2) There is an absence of modern research on substances gradually becoming more prevalent in the dry cleaning industry, particularly those hailed as promising alternatives. Prime examples include those alternatives evaluated by TURI, as discussed in Sect. 2, including high-flashpoint hydrocarbons, propylene glycol ethers, butylal, siloxane, and N-propyl bromide. This lack of published data is also highlighted by the numerous unassigned or unclassifiable IARC classifications (i.e., butylal, high-flashpoint hydrocarbons, nonane, decane, undecane, xylene, toluene), as well as missing OEL and BEI values.

Despite employing a broad search criterion to encompass all relevant dry cleaning solvent types, the predominant focus of the published papers within the last decade pertained to PCE (66%), in spite of the industry’s reduced reliance on the substance due to ongoing regulatory shifts aiming to restrict PCE utilization. This highlights a notable discrepancy between the dry cleaning industry’s trajectory and the current occupational exposure research focus. It is inevitable that PCE prevalence will diminish over time, as the EPA has proposed a 10-year phase-out plan of the substance in the industry [68]. In light of this, it is critical to determine the safety of solvents that may take its place. Achieving this necessitates studies emphasizing exposure quantification and biological assessment, focusing on biochemical alterations, cellular and molecular outcomes (e.g., DNA mutations, interference with protein synthesis, cellular apoptosis), physiological changes, immune response, neurological impairments, and potential carcinogenic effects.

Additionally, NIOSH’s Risk Management Limit for Carcinogens follows guidelines rooted in the principle that no level of exposure to identified carcinogenic substances is considered to be safe [69]. Given this stance, exposure to concentrations that exceed OEL or BEI values present significant health risks; even values under established exposure limits for PCE (Group 2A carcinogen), TCE (Group 1 carcinogen), TCA (Group 2B carcinogen), and Benzene (Group 1 carcinogen), do not indicate safety, but rather imply increased cancer risk. Further, several chemicals, including butylal, high-flashpoint hydrocarbons, nonane, decane, and undecane, are yet to receive IARC classifications, while xylenes and toluene are classified as Group 3, indicating insufficient carcinogenetic research. In this instance, when there is inadequate data, NIOSH and IARC follow the “As Low As Reasonably Achievable” (ALARA) premise, an inherently subjective guideline that leaves room for inconsistencies [70].

Studies that collected biological data, specifically those that reported biomarkers for exposure like Everatt et al. 2013 and Azimi et al. 2017, have demonstrated evidence of genotoxic effects amongst dry cleaners through statistically significant increases in MN frequency and DNA damage compared to controls [65, 66]. Techniques like the comet assay and analysis of MN and CA are routinely utilized to monitor genetic damage in humans. These methods offer valuable insights into biomarker exposure effects, with abnormal readings associated with early events in carcinogenesis [71–74]. Everatt et al. 2013 showcased the quality of these findings through multi-type analysis, blind evaluation by three scorers for unbiased biomarkers assessment, and extensive statistical methods, while Azimi et al. 2017 implemented meticulous comet assay procedures, blind evaluation for objective image analysis and thorough statistical testing methods. Biological effects were observed even at PCE concentrations below the established OELs, evidence that current limits may not adequately protect against genotoxic damage and that more stringent OELs may benefit workers’ health. This observation reinforces NIOSH’s guidelines that no safe level of exposure exists for carcinogenic substances.

The two studies examining TCE and one examining benzene exposure provided exposure-related insights. Friesen et al.’s 2015 retrospective survey revealed high TCE concentrations in the late 1970s, surpassing established exposure limits. However, these measurements were taken before exposure regulations were in place. In Sadeghi et al.’s 2013 more recent study, TCE concentrations varied, with the grand mean falling within acceptable limits but with sporadic instances of high exposure. Although neither study explored the health effects of TCE exposure, TCE, like benzene, is classified as a Group 1 carcinogen, indicating its genotoxicity based on substantial human data. Therefore, there is definitively no safe level of exposure [5, 75]. Shim et al.'s 2013 study did, however, provide health-related data, reaffirming benzene exposure risks. Governments globally are moving to ban cancer-linked chemicals like benzene and TCE from the dry cleaning industry. As a result, usage is expected to decline over time. However, monitoring is still necessary for those facilities that use these substances to ensure exposures do not surpass OELs; even sporadic instances of high exposure can have detrimental health effects, highlighting the ongoing need for preventive measures.

The available literature on solvents used in the laundry and dry cleaning industry reveals a significant gap in research pertaining to alternative solvents to PCE. Among the three published studies (Modenese et al. 2019, Ceballos et al. 2016, & Eun et al. 2022) exploring less studied but still widely used solvents, TCA, butylal, high-flashpoint hydrocarbons, and one study examining various VOCs (nonane, decane, undecane, xylenes, & toluene), all study designs failed to monitor exposure related health outcomes. This lack of data regarding the health effects of alternative solvents is concerning, especially considering the limited availability of OELs for these substances. Currently, only nonane, o-xylene, and toluene have established OELs, and TCA has a BEI for urinary levels, of which all samples fell within standard ranges [54, 76–78].

Examining human health effects is crucial when investigating newly introduced solvents, as it provides insights into immediate and long-term health impacts and assists in formulating informed decisions and guidelines to keep workers safe. The scarcity of this type of published literature plays a significant role in the absence of established OELs for these solvents. Further, this present limitation has additionally contributed to the lack of IARC carcinogenicity classification for these solvents, with only TCA, xylenes, and toluene having been classified as Group 2B (possibly carcinogenic) and Group 3 (uncertain carcinogenicity) by IARC, respectively [79–81]. Consequently, dry cleaning workers face exposure to solvents that pose unknown health risks without clear information on their carcinogenic impact or the specific cancers associated with them. Compounding this issue is the unavailability of accessible information about the effects of exposure to these solvents. The resources expected to provide insights, such as agencies responsible for establishing OELs and evaluating carcinogenicity levels, lack the necessary data to inform workers about these critical concerns.

## Conclusion

The presented scoping review analyzed 12 studies relevant to the laundry and dry cleaning industry and their examined solvent types. Reported exposure values were compared to established OELs and BEIs, as well as their designated IARC classification, if available. Despite comprehensive search criteria and significant diversity across study methodologies, 66% of the included studies failed to investigate exposure-related health outcomes. All studies investigating emerging dry cleaning solvents (alternatives to PCE), which presently lack human carcinogenicity data, did not measure exposure biomarkers that would be indicative of carcinogenic potential. This information gap is further accentuated by the unassigned carcinogenic classification for many of these solvents. As regulatory changes further restrict PCE use, understanding the safety profile of alternate solvent types is essential to avoid latent health risks.

Agencies like NIOSH emphasize that no level of carcinogenic exposure is safe. However, many solvents in use or under consideration lack the proper research to determine carcinogenic risk. This scarcity of such research deters the establishment of more accurate OELs, possibly exposing dry-cleaning workers to unidentified health risks. Further, two studies found a potential association between PCE exposure and DNA damage among dry cleaners, even at levels below OEL, hinting at a possible need to reconsider these thresholds and supporting that no carcinogen exposure is acceptable.

Future studies should prioritize investigating the potential health effects and carcinogenic properties of exposure to new and alternative solvents, such as butylal and high-flashpoint hydrocarbons. These two substances lack OELs, BEIs, and IARC classifications. Transformation and genotoxicity assay studies are crucial first steps that need to be taken to assess the potential carcinogenicity of exposure to these emerging and supposedly greener alternative chemicals in the dry cleaning industry. These in vitro studies will help determine if there is a causal relationship between solvent exposure and cancer development as new solvents become more widely used. In addition, large-scale observational studies that monitor exposure levels and health effects should be conducted at facilities already utilizing alternative solvents to PCE to close the current research gap.

While this scoping review primarily aims to identify gaps in the existing literature concerning exposure risks and associated health outcomes in the dry cleaning industry, the implications of these findings extend beyond this specific industry to other industries and the general population engaging with solvents. Most individuals conduct laundry activities and may inadvertently expose themselves to harmful solvents commonly used in dry cleaning. Therefore, the recommendations derived from this review can benefit those working in the dry cleaning industry and potentially safeguard the general public’s health.

### Electronic supplementary material

Below is the link to the electronic supplementary material.

Supplementary Material 1. The supplementary Information (SI) document contains VOC background information and tables regarding the used inclusion and exclusion criteria (SI Table SI), search terms (SI Table S2), articles returned for search by database (SI Table S3), and the final retrieved scoping review articles (SI Table S4)

## Data Availability

All data analyzed during this study are included in this published article. For further inquiries regarding data, contact the corresponding author.

## List of abbreviations

ILO: International Labour Organization
VOC: Volatile organic compounds
OEL: Occupational exposure limits
TCE: Trichloroethylene
PCE: Perchloroethylene
SMR: Standardized Mortality Ratio
NIOSH: National Institute for Occupational Safety and Health
SMR: Standardized mortality ratio
CI: Confidence interval
IARC: International Agency for Research on Cancer
OSHA: Occupational Safety and Health Administration
PEL: Permissible exposure limit
ppm: Parts per million
TWA: Time-weighted average
ACGIH: American Conference of Governmental Industrial Hygienists
TLV: Threshold limit value
STEL: Short-term exposure limit
REL: Recommended exposure limit
EPA: Environmental Protection Agency
REACH: Registration, Evaluation, Authorization, and Restriction of Chemicals
NICNAS: Australia’s National Industrial Chemicals Notification and Assessment Scheme
TURI: Toxic Use Reduction Institute
HAPs: Hazardous air pollutants
ODS: Ozone-depleting substances
PGE: Propylene Glycol Ethers
DPnB: Glycol n-butyl
DPtB: Dipropylene glycol tert-butyl
n-PB: n-Propyl Bromide
REACH: Registration, Evaluation, Authorization, and Restriction of Chemicals
PRISMA-ScR: Preferred Reporting Items for Systematic Reviews and Meta-Analyses- Scoping Reviews
BEI: Biological Exposure Indices
TL: Tail length
TM: Tail moment
CA: Chromosome aberration
MN: Micronuclei
TCA: Trichloroacetic acid
IRC: Inhalation reference concentration
SOAP: Secondary organic aerosol potential
POCP: Photochemical ozone creation penitential
ALARA: As Low As Reasonably Achievable
SD: Standard deviation
GM: Geometric mean
GSD: Geometric standard deviation
