# Supplementary material for: Occupational exposures and cancer risk in commercial laundry and dry cleaning industries: a scoping review
Source: BMC Public Health. 2023 Dec 21;23:2561. doi: 10.1186/s12889-023-17306-y (PMC10740271; doi:10.1186/s12889-023-17306-y)
Supplement: Supplementary file 1 — Supplementary Material 1. The supplementary Information (SI) document contains VOC background information and tables regarding the used inclusion and exclusion criteria (SI Table SI), search terms (SI Table S2), articles returned for search by database (SI Table S3), and the final retrieved scoping review articles (SI Table S4) [file 12889_2023_17306_MOESM1_ESM.docx]

**Supporting Information**

**Occupational Exposures in Commercial Laundry and Dry Cleaning Industries and their Associative Cancers: A Scoping Review**

Emma Ann Landskroner ^1^ Candace Su-Jung Tsai ^2*^

^1, 2*^ Department of Environmental Health Sciences, Fielding School of Public Health, University of California Los Angeles, Los Angeles, CA

*Corresponding author: Dr. Candace S.J. Tsai,

Email: candacetsai@ucla.edu

Affiliation: Department of Environmental Health Sciences, Fielding School of Public Health, University of California, Los Angeles, Los Angeles, California, 90095-1735, United States

Address: 650 Charles E. Young Drive S., MC 177220, Los Angeles, California 90095-1735, United States

Phone: (310) 206-9258

**TABLE OF CONTENT**

**VOC Section.** Background information on volatile organic compounds

**Table S1**. Inclusion and exclusion criteria

**Table S2**. Search terms

**Table S3**. Articles returned for search by database

**Table S4.** Examined substances and their associated occupational exposure limits

**References.** Articles cited

**SUPPORTING INFORMATION**

**VOC Section.**

Volatile organic compounds (VOCs) are organic chemicals that have high vapor pressure at room temperature, low water solubility, a boiling point less than or equal to 482 °F (250 °C), and are emitted as gases (1, 2, 3). They can undergo photochemical reactions, forming secondary organic aerosols, and contain hazardous carcinogenic compounds that can be released from various sources, including cleaning agents, solvents, pesticides, and paints (4, 5).

In dry cleaning, using certain solvents leads to the emission of VOCs (6, 7, 8). Studies have found that significant and hazardous VOC emission occurs from petroleum-based solvents via machine exhaust gas (2, 9, 10, 11). Common solvents such as PCE, TCE, hydrocarbons, toluene, and xylene are classified as VOCs and can potentially cause cancer at high concentrations. Although, only a few VOCs, such as formaldehyde and benzene, have been officially considered carcinogenic to humans (Group 1) by the International Agency for Research on Cancer (IARC) (12, 13, 14). Individuals who work with VOCs indoors, such as dry cleaners, face a greater risk of higher VOC exposure, as indoor concentrations can be up to ten times greater than in outdoor environments, increasing the likelihood of chronic exposure (7).

**Table S1**. List of inclusion and exclusion criteria by publication type, study type, population of interest, language, publication time, exposure of interest, and sampling.

|  | **Inclusion Criteria** | **Exclusion Criteria** |
| --- | --- | --- |
| **Publication Type** | Peer reviewed | Non-peer reviewed |
| **Study Type** | Cohort studies, cross-sectional studies, case studies, case reports, biomonitoring studies, risk assessment studies, retrospective studies | Overview, commentary, editorial, or opinion articles |
| **Population** | Must include workers in the laundry and dry cleaning industry | Workers outside of specified occupation |
| **Language** | English | Studies not published in English |
| **Publication Time** | January 1, 2012 – December 1, 2022 | Published outside of January 1, 2012 – December 1, 2022 |
| **Exposure** | Must identify exposure | Does not identify exposure |
| **Sampling** | Must conduct sampling | Does not conduct sampling |

**Table S2**. List of search terms used by relevant occupational terms, exposure of interest terms, and outcome of interest term.

| **Occupational Search Terms** | **Exposure of Interest Terms** | **Outcome of Interest Term** |
| --- | --- | --- |
| Laundry service* OR Laundry workers*OR Laundry attendant* OR Laundromat* OR Dry cleaning service* OR Dry cleaning workers* OR Dry cleaning attendant | AND Laundry solvent* OR Dry cleaning solvent* OR N-Propyl bromide* NPB* OR 1-Bromopropane* OR Tetrachloroethylene* OR TCE* OR Perchloroethylene* OR PCE* OR Benzene* OR Acetone* OR Toluene* OR Ethylbenzene* OR Xylene* OR Chloroform* OR Formaldehyde* OR Butylal* OR Butanol* | AND Cancer* |

**Table S3.** Articles identified from search by database.

| **Database** | **Number of Articles Returned for Search** |
| --- | --- |
| PUBMED | 212 |
| Science Direct | 153 |
| NIH | 68 |
| Embase | 11 |
| EBSCOhost | 19 |
| Google Scholar | 5 |
| Total Articles Returned for Search = 468 | |

**Table S4.** A list of all substances examined and their OSHA, ACGIH, and NIOSH occupational exposure limits, including ACGIH BEI values.

| **Substances** | **OSHA OEL** | **NIOSH OEL** | **ACGIH OEL** | **ACGIH BEI** |
| --- | --- | --- | --- | --- |
| PCE | **PEL-TWA** (8hr.) 100ppm (678 mg/m^3^) (26) | Lowest feasible concentration (26) | **TLV-TWA** (8hr.) 25ppm (170 mg/m^3^)  **TLV-STEL** (15min.) 100ppm (685 mg/m^3^) (27) | **In end-exhaled air:** Prior to shift 3ppm (20.34 mg/m3)  **In blood**: Prior to shift 0.5 mg/L (27) |
| TCE | **PEL-TWA** (8hr.) 100ppm (535 mg/m^3^) (28) | **REL-TWA** (10hr.) 25ppm (134.3 mg/m^3^) (28) | **TLV-TWA** (8hr.) 10ppm (54 mg/m^3^)  **TLV-STEL** (15min.) 25ppm (135 mg/m^3^) (27) | **In urine:** End of workweek 15mg/L  **In blood**: End of workweek 0.5 mg/L (27) |
| TCA | NA | **REL-TWA** (10hr.) 1ppm (6.68 mg/m^3^) (29) | **TLV-TWA** (8hr.) 0.5 ppm (3.34 mg/m^3^)  (27) | **In urine:** End of workweek 15mg/L  (27) |
| Benzene | **PEL-TWA** (8hr.) 1ppm (3.19 mg/m^3^)  **PEL-STEL** (15min.) 5ppm (15.97 mg/m^3^) (30) | **REL-TWA** (10hr.) 0.1 ppm 1ppm (0.32 mg/m^3^) (31) | **TLV-TWA** (8hr.) 0.02 ppm (0.0639 mg/m^3^)  **TLV-STEL** (15min.) 0.1ppm (0.32 mg/m^3^) (27) | **In urine:** End of shift 500 μg/g creatinine (27) |
| Butylal | NA | NA | NA | NA |
| High-Flashpoint Hydrocarbon | NA | NA | NA | NA |
| Nonane | NA | **REL-TWA** (10hr.) 200ppm (1050 mg/m^3^) (32) | **TLV-TWA** (8hr.) 200ppm (1050 mg/m^3^) (27) | NA |
| Decane | NA | NA | NA | NA |
| Undecane | NA | NA | NA | NA |
| O-xylene | **PEL-TWA** (8hr.) 100ppm (435 mg/m^3^) (33) | **REL-TWA** (10hr.) 100ppm (435 mg/m^3^)  **REL-STEL** (15min.) 150ppm (655 mg/m^3^) (33) | **TLV-TWA** (8hr.) 20ppm (86.83 mg/m^3^) (27) | **In urine:** End of shift 1.5 g/g creatinine (27) |
| Toluene | **PEL-TWA** (8hr.) 200ppm (750 mg/m^3^) (34) | **REL-TWA** (10hr.) 100ppm (375 mg/m^3^)  **REL-STEL** (15min.) 150ppm (560 mg/m^3^) (34) | **TLV-TWA** (8hr.) 20ppm (75.33 mg/m^3^) (27) | **In urine:** End of shift 0.03 mg/L  **In blood**: Prior to last shift of workweek 0.02 mg/L (27) |

**References:**

1. What are volatile organic compounds (VOCs)? [Website]. United States Environmental Protection Agency website: EPA; 2023 [updated March 15, 2023. Available from: <https://www.epa.gov/indoor-air-quality-iaq/what-are-volatile-organic-compounds-vocs>.

2. Lee H, Kim K, Choi Y, Kim D. Emissions of Volatile Organic Compounds (VOCs) from an Open-Circuit Dry Cleaning Machine Using a Petroleum-Based Organic Solvent: Implications for Impacts on Air Quality. Atmosphere. 2021;12(5):637.

3. Indoor Air Quality (IAQ) Technical Overview of Volatile Organic Compounds [Website ]. United States Environmental Protection Agency; 2023 [updated March 14, 2023. Available from: <https://www.epa.gov/indoor-air-quality-iaq/technical-overview-volatile-organic-compounds>.

4. Eun D-M, Han Y-S, Park S-H, Yoo H-S, Le YT-H, Jeong S, et al. Analysis of VOCs Emitted from Small Laundry Facilities: Contributions to Ozone and Secondary Aerosol Formation and Human Risk Assessment. International Journal of Environmental Research and Public Health. 2022;19(22):15130.

5. Srivastava D, Vu TV, Tong S, Shi Z, Harrison RM. Formation of secondary organic aerosols from anthropogenic precursors in laboratory studies. npj Climate and Atmospheric Science. 2022;5(1).

6. Ruder AM, Ward EM, Brown DP. Mortality in dry-cleaning workers: an update. Am J Ind Med. 2001;39(2):121-32.

7. David E, Niculescu V-C. Volatile Organic Compounds (VOCs) as Environmental Pollutants: Occurrence and Mitigation Using Nanomaterials. International Journal of Environmental Research and Public Health [Internet]. 2021; 18(24).

8. Çankaya S, Pekey H, Pekey B, Özerkan Aydın B. Volatile organic compound concentrations and their health risks in various workplace microenvironments. Human and Ecological Risk Assessment: An International Journal. 2020;26(3):822-42.

9. Song M, Kim K, Cho C, Kim D. Reduction of Volatile Organic Compounds (VOCs) Emissions from Laundry Dry-Cleaning by an Integrated Treatment Process of Condensation and Adsorption. Processes. 2021;9(9):1658.

10. Goodman NB, Wheeler AJ, Paevere PJ, Agosti G, Nematollahi N, Steinemann A. Emissions from dryer vents during use of fragranced and fragrance-free laundry products. Air Quality, Atmosphere & Health. 2019;12(3):289-95.

11. Guo H, Lee SC, Chan LY, Li WM. Risk assessment of exposure to volatile organic compounds in different indoor environments. Environ Res. 2004;94(1):57-66.

12. Wallace LA. ASSESSING HUMAN EXPOSURE TO VOLATILE ORGANIC COMPOUNDS. In: Spengler JD, Samet JM, McCarthy JF, editors. Indoor Air Quality Handbook. 1st Edition ed. New York: McGraw-Hill Education; 2001.

13. Safety and Health Topics [Website]. United States Department of Labor Occupational Safety and Health Administration [Available from: <https://www.osha.gov/formaldehyde/hazards#:~:text=Organization%20(WHO).-,Formaldehyde.,to%20humans%20(Group%201>).

14. Benzene and Cancer Risk [Website]. American Cancer Society; 2023 [updated February 1, 2023. Available from: <https://www.cancer.org/healthy/cancer-causes/chemicals/benzene.html#:~:text=IARC%20classifies%20benzene%20as%20%E2%80%9Ccarcinogenic,%2C%20and%20non%2DHodgkin%20lymphoma>.

15. Ceballos DM, Whittaker SG, Lee EG, Roberts J, Streicher R, Nourian F, et al. Occupational exposures to new dry cleaning solvents: High-flashpoint hydrocarbons and butylal. Journal of Occupational and Environmental Hygiene. 2016;13(10):759-69.

16. Friesen MC, Locke SJ, Chen YC, Coble JB, Stewart PA, Ji BT, et al. Historical occupational trichloroethylene air concentrations based on inspection measurements from Shanghai, China. Ann Occup Hyg. 2015;59(1):62-78.

17. Habib S, Ahmed HO, Al-Muhairi N, Ziad R. Preliminary Study: Environmental Assessment of Perchloroethylene in Dry-Cleaning Facilities in the UAE. J Environ Public Health. 2018;2018:1732906.

18. Sadeghi M, Nadafi K, Nabizadeh R, Nasseri S, Mesdaghinia A, Mahvi AH, et al. Perchloroethylene and Trichloroethylene in the Air and Effluent of Dry Cleaning Shops. International Journal of Occupational Hygiene 2015;6(1):11-5.

19. Dias CM, Menezes HC, Cardeal ZL. Use of exhaled air as an improved biomonitoring method to assess perchloroethylene short-term exposure. Environmental research. 2017;156:108-12.

20. Everatt R, Slapšytė G, Mierauskienė J, Dedonytė V, Bakienė L. Biomonitoring study of dry cleaning workers using cytogenetic tests and the comet assay. J Occup Environ Hyg. 2013;10(11):609-21.

21. Lucas D, Hervé A, Lucas R, Cabioch C, Capellmann P, Nicolas A, et al. Assessment of Exposure to Perchloroethylene and its Clinical Repercussions for 50 Dry-Cleaning Employees. J Occup Environ Hyg. 2015;12(11):767-73.

22. Modenese A, Gioia TC, Chiesi A, Abbacchini C, Borsari L, Ferrari D, et al. Evaluation of Occupational Exposure to Perchlorethylene in a Group of Italian Dry Cleaners Using Noninvasive Exposure Indices. International Journal of Environmental Research and Public Health. 2019;16(16):2832.

23. Azimi M, Bahrami MR, Rezaei Hachesu V, Zavar Reza J, Mihanpour H, Zare Sakhvidi MJ, et al. Primary DNA Damage in Dry Cleaners with Perchlorethylene Exposure. Int J Occup Environ Med. 2017;8(4):224-31.

24. Shim KY, Cha SW, Um WH, Chun CG, Jeong SW, Jang JY, et al. Simultaneous occurrence of gallbladder cancer in a laundry couple: association between gallbladder cancer and benzene. Korean J Gastroenterol. 2013;61(2):107-9.

25. Ziener C-E, Braunsdorf P-P. Trace Analysis in End-Exhaled Air Using Direct Solvent Extraction in Gas Sampling Tubes: Tetrachloroethene in Workers as an Example. International Journal of Analytical Chemistry. 2014;2014:1-10.

26. OSHA Occupational Chemical Database [Website]. United States Department of Labor: Occupational Safety and Health Administration; 2022 [updated June 6, 2022. Available from: <https://www.osha.gov/chemicaldata/190>.

27. 2023 TLVs and BEIs Based on the Documentation of the Threshold Limit Values for Chemical Substances and Physical Agents & Biological Exposure Indices Cincinnati, OH: American Conference of Governmental Industrial Hygienists; 2023.

28. OSHA Occupational Chemical Database [Website]. United States Department of Labor: Occupational Safety and Health Administration; 2021 [updated April 14, 2021. Available from: <https://www.osha.gov/chemicaldata/684>.

29. NIOSH Pocket Guide to Chemical Hazards Center for Disease Control and Prevention: The National Institute for Occupational Safety and Health (NIOSH); 2019 [updated October 30, 2019. Available from: <https://www.cdc.gov/niosh/npg/npgd0626.html>.

30. OSHA Occupational Chemical Database United States Department of Labor: Occupational Safety and Health Administration; 2021 [updated April 6, 2021. Available from: <https://www.osha.gov/chemicaldata/491>.

31. NIOSH Pocket Guide to Chemical Hazards: National Institute of Occupational Safety and Health 2019 [updated October 30, 2019. Available from: <https://www.cdc.gov/niosh/npg/npgd0049.html>.

32. OSHA Occupational Chemical Database [Website]. United States Department of Labor: Occupational Safety and Health Administration; 2021 [updated April 13, 2021. Available from: <https://www.osha.gov/chemicaldata/131>.

33. NIOSH Pocket Guide to Chemical Hazards [Website]. National Institute of Occupational Safety and Health 2019 [updated October 30, 2019. Available from: <https://www.cdc.gov/niosh/npg/npgd0668.html>.

34. OSHA Occupational Chemical Database [Website]. United States Department of Labor: Occupational Safety and Health Administration; 2022 [updated June 3, 2023. Available from: <https://www.osha.gov/chemicaldata/89>.
